# Improving Reading and Eye Movement Control in Readers with Oculomotor and Visuo-Attentional Deficits

**DOI:** 10.3390/jemr18040025

**Published:** 2025-06-23

**Authors:** Stéphanie Ducrot, Bernard Lété, Marie Vernet, Delphine Massendari, Jérémy Danna

**Affiliations:** 1 Aix Marseille Univ, CNRS, LPL, Aix-en-Provence, France; mvernet@ch-digne.fr (M.V.); delphine.massendari@gmail.com (D.M.); 2 Institute for Language, Communication, and the Brain, Aix-Marseille Université, Aix-en-Provence, France; 3 Laboratoire d’Étude des Mécanismes Cognitifs, Université Lumière Lyon 2, Lyon, France; bernard.lete@univ-lyon2.fr; 4 Centre de jour enfants, Centre hospitalier de Digne-les-Bains, France; 5 CLLE, Université de Toulouse, CNRS, Toulouse, France; jeremy.danna@cnrs.fr

**Keywords:** reading strategies, saccadic computation, PVL saliency, remedial tool, oculomotor and visuo-attentional deficits, poor readers, inexperienced readers

## Abstract

The initial saccade of experienced readers tends to land halfway between the beginning and the middle of words, at a position originally referred to as the preferred viewing location (PVL). This study investigated whether a simple physical manipulation—namely, increasing the saliency (brightness or color) of the letter located at the PVL—can positively influence saccadic targeting strategies and optimize reading performance. An eye-movement experiment was conducted with 25 adults and 24 s graders performing a lexical decision task. Results showed that this manipulation had no effect on initial landing positions in proficient readers, who already landed most frequently at the PVL, suggesting that PVL saliency is irrelevant once automatized saccade targeting routines are established. In contrast, the manipulation shifted the peak of the landing site distribution toward the PVL for a cluster of readers with immature saccadic strategies (with low reading-level scores and ILPs close to the beginning of words), but only in the brightness condition, and had a more compelling effect in a cluster with oculomotor instability (with flattened and diffuse landing position curves along with oculomotor and visuo-attentional deficits). These findings suggest that guiding the eyes toward the PVL may offer a novel way to improve reading efficiency, particularly for individuals with oculomotor and visuo-attentional difficulties.

## 1. Introduction

In reading, the eyes execute a series of saccades to bring new words into foveal vision. In languages with interword spacing, saccades typically land within words following a consistent pattern: the distribution of landing sites is skewed, with a peak just to the left of the word center [1,2]. This central tendency is known as the preferred viewing location OK (PVL; [2]). The PVL represents a statistical norm, i.e., the location where fixations most frequently land across multiple instances of reading.

In contrast, the initial landing position (ILP) refers to the actual fixation location on a specific trial or word. While the PVL emerges from aggregated reading behavior, the ILP captures a single, moment-to-moment decision made by the oculomotor system. While the ILP typically aligns with the PVL in skilled readers, it is still susceptible to modulation by low-level visual factors, such as parafoveal word length, spacing, and visual salience [1,3,4,5,6]. It is generally accepted that typical oculomotor behavior is characterized by a narrower spread of ILPs around the PVL [7]. It is often assumed that the PVL reflects a strategy in which readers attempt to fixate the center of the word (e.g., [1,8]; but see [9]) and that deviations from this position result from systematic oculomotor undershoot errors [1,10,11]. However, several studies demonstrated that the typical left-of-center PVL observed in left-to-right reading may not be primarily due to oculomotor error but rather reflects a processing advantage at that position. As suggested by [12], it would not be surprising that multiple higher-order factors contribute to the processing advantage that drives readers to land left of center in sentence reading. These include the mechanisms underlying left-to-right attentional scanning during reading [2,13,14], the advantage of being closer to the initial letters of the word, which carry crucial lexical information [13,14,15], and the development of a rightward perceptual bias through reading experience [16].

### 1.1. Saccade-Targeting Strategies in Inexperienced and Poor Readers

Learning to read can be challenging for some children, requiring the development of both linguistic skills and cognitive control of saccadic eye movements. Both ILP and, more prominently, PVL can serve as markers of the progressive development of reading expertise, as their stabilization reflects increasing automaticity and efficiency in eye movement control during reading. Specifically, fixation durations decrease while both saccade length and the size of the perceptual span increase [17,18,19,20,21]. Between Grades 1 and 3, ILPs progressively shift toward the PVL [19,21,22,23]. The emergence of the PVL is considered a hallmark of reading expertise, facilitating the development of parallel letter processing and engaging the lexical route [24], which enables word identification through direct matching between the written word form and its stored visual representation. During the first years of formal reading instruction, the PVL adjusts in parallel with the development of an optimal lexical and visuo-attentional (VA) processing strategy for word reading, allowing rapid and low-effort access to word meaning. This oculomotor and lexical processing efficiency enables skilled readers to achieve an average reading rate of over four words per second. This further suggests that saccade-targeting strategies are acquired through reading experience and may reflect the predominant reading route the reader uses (i.e., lexical vs. sublexical; [25,26]). In sequential reading, fixations move gradually through the word from left to right, reflecting the serial processing of sublexical units. In contrast, reliance on the lexical route is associated with ILPs concentrated near the word center, indicating rapid orthographic-to-semantic access [25,26].

Several studies have demonstrated that poor readers and dyslexics display atypical eye movement patterns (e.g., [26,27,28,29]). These patterns include more and longer fixations, shorter saccade lengths, and a higher frequency of regressions [6,30,31], resembling those of beginning readers. Moreover, dyslexic readers have been shown to land closer to the beginning of words, suggesting an overreliance on serial decoding strategies [27,28,32]. This atypical eye movement behavior may compromise reading efficiency. Suboptimal landing positions reduce the amount of useful visual and linguistic information extracted during fixation and often result in less efficient subsequent fixations. These fixations are frequently shorter and more likely to fall within the same word, as corrective re-fixations are rapidly triggered based on low-level visual cues [26,27,33,34,35,36]. Moreover, lexical access processes are more likely to fail if the useful visual information about the shape and location of ensuing words is degraded or lacking [6]. It has also been reported that individuals with developmental dyslexia (DD) have difficulty in narrowing their focus of attention, hampering the exact planning of fine-tuned saccades (e.g., diffuse spread of ILPs, unexpected/atypical saccades, e.g., [37,38,39,40,41]; see [42] for a meta-analysis). In this context, ref. [43] reliably used eye-movement parameters to identify individuals with reading difficulties involving atypical oculomotor strategies. Similar machine-learning methods have also been successfully implemented to detect dyslexia from eye movement data (see [44,45,46,47,48,49]; for review, see [50]). While not all dyslexic children exhibit oculomotor impairments [27,51,52,53], it remains possible that a subset of children with DD experiences occasional deficits in oculomotor control [26,54,55,56]. Such oculomotor deficits may reflect additional, non-linguistic sources of difficulty that interact with phonological or orthographic impairments, potentially compounding reading difficulties. Consequently, oculomotor anomalies in this subgroup could constitute a secondary factor contributing to impaired reading performance. Previous research (e.g., [55,56,57,58]) suggests that such oculomotor deficits may originate from underlying visual-processing deficits, such as VA deficits [26,41,59,60,61,62,63]. Although these deficits are often subtle and difficult to quantify, exploring targeted interventions designed to address them is worthwhile [27]. In this context, oculomotor training programs may hold promise for improving reading performance in individuals presenting such impairments [26,37,64,65,66].

### 1.2. Improving Saccadic Eye Movements

Currently, most screening, intervention, and educational tools available to professionals primarily focus on addressing children’s oral language difficulties, particularly those related to phonological skill acquisition [67,68]. In this context, interventions for children with DD have concentrated on enhancing linguistic capabilities, including phonics, orthographic, and morphological instruction [69]. However, several studies have underscored the importance of considering VA and perceptual skills for both the early identification of potential learning difficulties and their remediation once established ([61,70]; see also [42], for a meta-analysis). These skills are closely related to oculomotor behavior during reading and can be objectively assessed through indicators such as the PVL and the ILP. In this regard, it has been demonstrated that reading performance can be enhanced through training of VA and perceptual abilities among school-aged children ([61,71,72,73,74,75]; see also [76] for a review on action video games). Improving current rehabilitation and educational tools by incorporating oculomotor and VA training could enable more individuals with reading difficulties to achieve proficiency in written language.

Like any motor task, eye movements can be trained for improved execution, and multiple studies in clinical settings report successful outcomes following training [77,78,79,80]. Ref. [25] demonstrated the effectiveness of an eye movement-guided therapy approach that stimulated both lexical and segmental reading procedures in patients with central dyslexia. All patients benefited from the intervention; the total reading time, the number of fixations needed to identify the target, and reading accuracy improved significantly (see also [81], in a patient with pure alexia, and [82], in patients with hemianopic alexia). Emerging studies also support the efficacy of eye-tracking-based training as a cognitive intervention for children with learning difficulties [24,83,84,85,86,87], as well as for typically developing school-aged children [78,88]. Eye movement training has been shown to improve reading-related oculomotor behavior [79,89] and enhance reading skills. Ref. [78] found a significant effect of rigorous training on visual saccadic skills and reading fluency in young children. Ref. [88] investigated the effects of in-school saccadic training on reading fluency and comprehension in 327 first- and second-grade students. They showed that participants with high needs (i.e., those with eye movement deficits) demonstrated the greatest improvements. The authors concluded that these improvements were likely due to the repetitive practice of reading-related eye movements, shifting visuospatial attention, and visual processing. These findings align with other studies showing that eye movement control and VA abilities in readers with DD can improve through targeted training and repeated exposure, resulting in better reading performance (e.g., increased accuracy, reduced reading time, and fewer errors; [64,83,90,91,92,93]). Of particular interest is the study by [94], which used an eye tracker to capture readers’ eye movements and provide real-time feedback on their fixation locations, in order to train inexperienced readers to better control their eye movements during reading. The study demonstrated that the training game had the desired effect on the ILP distribution, with the peak of the ILP distribution shifting towards the PVL in most participants, confirming that triggering saccades toward the PVL leads to better reading outcomes.

### 1.3. Present Study

Research in visual perception has demonstrated that certain elementary visual features, such as color, orientation, or curvature, can elicit automatic attentional capture, even in the presence of distractors. This “pop-out” effect reflects an automatic stimulus-driven allocation of visual attention [95,96,97].

Building on this principle, the present study investigates whether increasing the visual saliency of the character located at the preferred viewing location (PVL), via enhanced brightness or color contrast, can facilitate more accurate saccadic targeting during reading. A salient letter within a word may function as an attentional cue during saccade planning, thereby promoting more precise eye movements and optimizing the initial landing position (ILP) [98,99]. This type of manipulation could offer a novel, non-linguistic method for scaffolding the development of efficient oculomotor routines in reading.

In addition to evaluating the global impact of PVL saliency on reading performance, the study aims to explore the relationship between reading profiles and saccade-targeting strategies. We hypothesize that visual cues attracting attention toward the PVL will be particularly beneficial for readers who have not yet acquired stable and efficient eye movement patterns, as well as for those whose difficulties are partially rooted in oculomotor or VA deficits.

By guiding attentional and oculomotor processes in a bottom-up manner, PVL saliency may facilitate the use of the direct (lexical) reading pathway, leading to measurable improvements in reading speed and accuracy. In this context, including adult readers is particularly relevant: although they have well-developed lexical knowledge and extensive reading experience, some may still exhibit residual oculomotor and VA deficits that limit reading efficiency. This population, therefore, offers the possibility to test the relevance of this strategy in the absence of linguistic difficulties or reading delays. Ultimately, this approach may serve both as a diagnostic probe for identifying inefficient saccadic strategies and as a targeted intervention tool to enhance reading fluency.

## 2. Method

### 2.1. Participants

Twenty-four typically developing children (13 females, mean age = 7.7 years, range = 7–8) and 25 adults (17 females, mean age = 20.6 years, age range of 18 to 25 years) participated in the study. They were native speakers of French, right-handed, and had normal or corrected-to-normal vision; none suffered from any neurological, psychiatric, or emotional disorders or were educationally disadvantaged. The adult participants were all students at Aix-Marseille University (France). The children were recruited in two elementary schools in Aix-en-Provence, a city in southern France, with their parents’ informed consent and the agreement of the board of education. The study received approval from the French Ethics Committee Review Board (2018-03-07-09) and followed the guidelines in the Declaration of Helsinki [100].

### 2.2. Material

*Lexical decision task (LDT).* The material comprises 120 words and 120 pseudowords, each 5 to 6 letters in length. Words were selected from the first-grade lemma lexicon of the Manulex database [101]. To maintain a moderate level of difficulty, half of the word stimuli were high-frequency items (mean printed frequency: 419 occurrences per million), while the other half were low-frequency items (mean printed frequency: 16 occurrences per million). All stimuli were displayed in light gray <COLOR 216 216 216>. The PVL saliency was manipulated by presenting either a colored <COLOR 0 255 0>, brighter (The brightness manipulation was intended to be imperceptible to the naked eye, although no psychophysical assessment was conducted to verify this. However, we asked participants at the end of the experiment whether they had noticed anything besides the letter colored in green, and 97% of them responded no. Only two participants perceived the presence of a brighter letter than the others) <COLOR 255 255 255>, or a neutral <216 216 216> letter at the PVL. Stimuli were displayed in the RVF, relative to the initial fixation point. The 240 items were divided into three lists (i.e., 80 items per list), with the items presented in a randomized order within each list for each participant. Each participant was exposed to the three PVL saliency conditions (neutral, brightness, colored), with one list per condition. The assignment of PVL saliency conditions to lists was counterbalanced across participants, such that each list appeared equally often in each condition across the sample. In addition, the order in which participants were exposed to the three PVL saliency conditions was also counterbalanced, so that each condition appeared equally often in each serial position (first, second, or third).

*Visual-processing skills assessment.* Visual-attentional (VA) skills were assessed using the Developmental Eye Movement (DEM) test ([102], a standardized tool frequently used in clinical and research contexts. The test requires participants to read aloud sequences of digits displayed in vertical (Tests A and B) and horizontal (Test C) arrangements across three separate sheets. Participants were instructed to read as quickly and accurately as possible. Three main indices were derived: (1) Vertical Time (VT), reflecting the total time (in seconds) to read vertically arranged digits in Tests A and B; (2) Adjusted Horizontal Time (HTaj), measuring the time (in seconds) to read the horizontally arranged digits in Test C, corrected for errors; and (3) Number of Errors, indicating the accuracy of performance on Test C. According to [102], VT primarily reflects automaticity skills, while HT provides a composite measure of both automaticity and oculomotor control relevant to reading. HT is particularly informative because it approximates the visuo-attentional and motor demands of reading, including systematic left-to-right scanning, return sweeps, and the execution of saccades of varying amplitudes [103].

*Reading skills assessment.* Reading efficiency was evaluated using the *Alouette* test [104,105], a widely used standardized French assessment. Participants were instructed to read aloud a 265-word passage as quickly and accurately as possible within a three-minute time limit. The text consists of syntactically correct but semantically impoverished sentences, minimizing contextual cues and allowing for a focused assessment of decoding skills only. The test provides an index of reading fluency but does not evaluate comprehension.

### 2.3. Apparatus and Procedure

Participants were seen individually during two sessions of approximately 30 min. The first one included the evaluation of reading efficiency with the *Alouette* test and VA skills with the DEM test. The second one included the lateralized lexical decision task. All participants were tested in a quiet, separate room. The lighting in the room was adjusted to a comfortable level for each participant and each task.

The procedure followed that of [19]. Eye movements during the lateralized lexical decision task were recorded using a mobile infrared, head-mounted eye-tracking system (Eyelink II, SR Research Ltd., Canada). The system monitored the right eye at a sampling rate of 250 Hz, with a spatial resolution of less than 0.04°, relying on infrared light reflections from the pupil and cornea. Participants’ head movements were minimized using a combined chin and forehead rest. A standard 9-point calibration procedure was carried out across the entire display area prior to the task. Eye-tracking data acquisition and stimulus presentation were managed through a Dell Latitude D600 laptop connected via a Dell D-type docking station. Visual stimuli were presented in lowercase Courier New font (22 pt) on a black background, using a 14-inch color monitor with a screen resolution of 1400 × 1050 pixels. The font color of the letter located at the PVL varied depending on the PVL saliency condition and was either light gray (neutral), white (brightness), or green (colored). Participants were seated in front of the screen at 60 cm. Participants were positioned 60 cm from the screen. At this viewing distance, each character subtended approximately 0.38° of visual angle, and 1° corresponded to 0.95 cm. Inter-letter spacing was 1 mm, equivalent to 2.8 points or 0.09° of visual angle.

**Figure 1:**
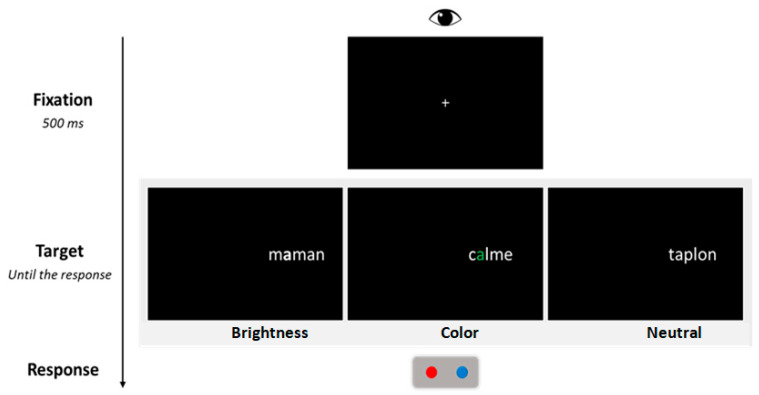
The procedure used in the lateralized lexical decision task. The figure illustrates the sequence of events within a trial, as well as the three display conditions corresponding to the saliency manipulation (brightness, color, and neutral).

Each trial followed a fixed sequence of events (see Figure 1). Participants were required to maintain fixation on the central cross displayed at the center of the screen. The experimenter provided continuous reminders to ensure that participants kept their gaze fixed and refrained from shifting their eyes away from the fixation point. After 500 ms, the central fixation cross was replaced by a parafoveal target displayed in the RVF. The nearest character in the stimulus was positioned 2.5 characters away from the fixation point. Participants were then required to decide as quickly and accurately as possible whether the stimulus was a French word and to press the corresponding button (blue button for “yes”, red button for “no”, see Figure 1). After the response, the screen was cleared for the next trial that started 500 ms later. Participants performed 12 practice trials prior to the experimental session.

### 2.4. Data Analysis

The eye-tracking data were analyzed using customized software scripts written in C++03 (Emaa software package: [106]). Fixations and saccades interrupted by blinks were excluded from further analysis. Fixations less than 80 ms were also deleted from the data set. In addition, 8.39% of the trials were discarded because of a lack of eye movement, an initial saccade triggered in the wrong direction, or a change in the recorded position of the eye while the participant was looking at the fixation point (since a head movement was suspected in this case). The data exclusion was independent of the experimental conditions. The following measures were computed (The data presented pertain to words only. A pilot study using the same task without eye-tracking, conducted with 150 students from first to fifth grade, showed that the saliency effect—when present—was limited to words. Follow-up analyses will be conducted to further examine the relationship between the saliency effect and lexicality): (1) the initial landing position (in characters), (2) the number of fixations, (3) the percentage of correct responses, and (4) the reaction time (in milliseconds, computed for correct responses only) (Table 1). The means were calculated for each participant in each condition for all measured variables.

### 2.5. Statistical Analysis

**Figure 2:**
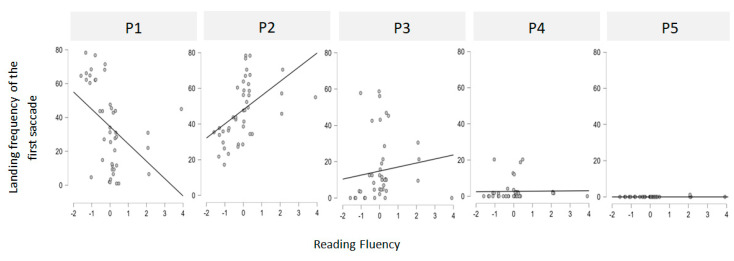
Scatterplots showing the correlations between the Reading Fluency index (from the *Alouette* test) and the initial landing position (ILP) index for each of the five landing sites.

Correlational analyses were first conducted to examine the relationship between reading fluency and landing preferences. Each participant was assigned (1) an initial landing position (ILP) index, defined as the most frequent initial fixation location on words presented in the right visual field (RVF), coded from position 1 (P1, leftmost zone) to position 5 (P5, rightmost zone), with an ILP at P2 corresponding to the PVL (Each stimulus was divided into five equally wide zones (i.e., one letter wide for a five-letter stimulus and 1.2 letters wide for a six-letter stimulus)); and (2) a Reading Fluency index, derived from each participant’s performance on the *Alouette* reading test. The correlations between these two measures are shown in Figure 2. A significant positive correlation was found between reading fluency and the probability of initially landing at position P2 [r(42) = 0.429, *p* = 0.001], suggesting that more fluent readers tend to target the PVL. In contrast, a negative correlation was found between reading fluency and the probability of landing at P1 [r(42) = –0.479, *p* = 0.005], indicating that less fluent readers tend to undershoot the PVL and fixate closer to the word onset. No significant correlations were found for positions 3, 4, or 5 (all *p* > 0.05). These results support the hypothesis that ILP variability reflects individual differences in oculomotor efficiency and reading expertise.

**Figure 3:**
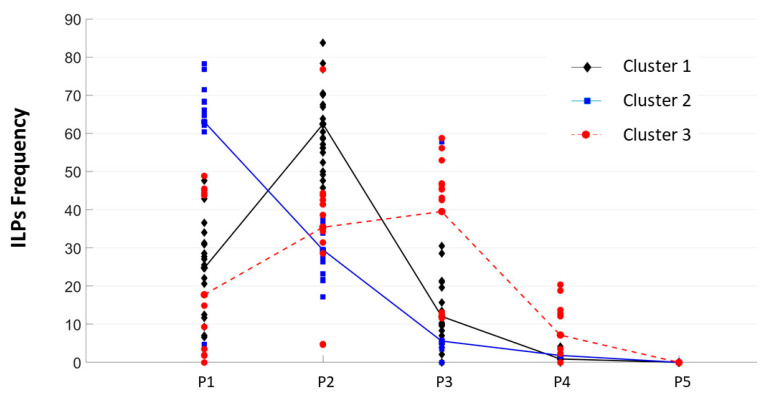
Individual distributions of the initial landing position’s frequency for words in the RVF, expressed as percentages. Each dot represents one participant’s ILP distribution, with color coding indicating cluster membership (Cluster 1 = black, Cluster 2 = blue, Cluster 3 = red).

A cluster analysis was then conducted based on ILP frequency at positions P1, P2, and P3 in order to further investigate individual differences in ILP patterns, following the methodology described by [27]. ILPs were analyzed using interactive partitioning (K-means clustering), aiming to minimize within-cluster variability and maximize between-cluster separation. A three-cluster solution was retained. The resulting clusters reflected distinct saccade-targeting strategies associated with different reading profiles, as previously demonstrated by [107] and more recently by [27]. Figure 3 presents the individual distributions of ILP frequencies across positions for each of the three participant clusters.

The first cluster, which included nearly half of the sample (49%, *N* = 23), was composed predominantly of participants without identified reading (RD) or visuo-attentional (VA) difficulties. These individuals exhibited an ILP consistently centered on the PVL (i.e., P2), reflecting a reading strategy characteristic of skilled readers. In contrast, Cluster 2 (27%, *N* = 14), mainly composed of participants with low reading proficiency, showed a tendency to fixate closer to the beginning of words (P1), a pattern often observed in developing or less efficient readers. This group had the lowest average score on the Reading Fluency index (M = −1.26 SD), suggesting reliance on a more sequential or letter-by-letter decoding strategy. Cluster 3 (25%, *N* = 12) was primarily composed of participants with visuo-attentional deficits. Their ILP distribution was more diffuse, spanning a broader area around the word center, and did not exhibit a clear peak. This pattern, previously interpreted as reflecting oculomotor instability [108] was supported by their low performance on the DEM-test (VT index: M = −1.41 SD; HTaj index: M = −1.94 SD), with 42% and 75% of participants scoring below −1.5 SD on these respective measures. Statistical analyses confirmed that the three clusters differed (Considering the chronological age of children’s participants, the 3 clusters did not differ significantly, F < 1) significantly in reading fluency [F(2,40) = 16.94, *p* < 0.001, *np*^2^ = 0.459] and in visuo-attentional skills [VT: F(2,46) = 25.92, *p* < 0.001, *np*^2^ = 0.530; HTaj: F(2,46) = 23.7, *p* < 0.001, *np*^2^ = 0.507]. The pairwise comparisons indicated that participants in Cluster 2 had significantly lower fluency scores than those in Clusters 1 (*p* < 0.001) and 3 (*p* < 0.01). Although participants in Cluster 3 showed lower performance on the Reading Fluency index than those in Cluster 1, the difference did not reach statistical significance (*p* > 10). Pairwise comparisons also revealed better VA skills in Cluster 1 and Cluster 2 than in Cluster 3 (both *p*s < 0.001) and no difference between Cluster 1 and Cluster 2 in terms of DEM scores (all *p* > 0.10).

We then investigated the extent to which the visual saliency of the letter located at the PVL influences eye behavior (initial landing position, Table 2) and reading efficiency (mean number of fixations reading reaction time and accuracy, Table 3). For all the following variables, analyses of variance (ANOVAs) were conducted, based on the Cluster mean performance, using the following design: Saliency (3 types of saliency: neutral, color, and brightness) × Position (5 modalities: P1, P2, P3, P4, and P5).

### 2.6. Initial Landing Positions

**Figure 4:**
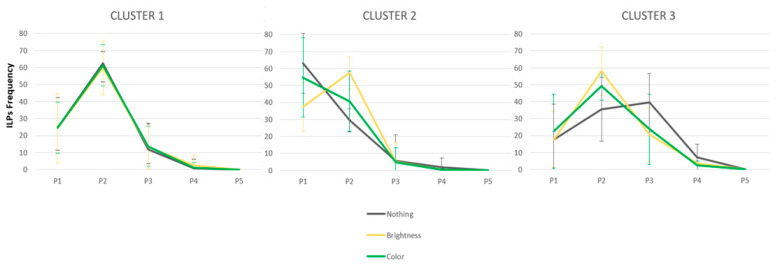
Distributions of the initial landing position’s frequency (in percentage) as a function of Cluster and PVL saliency.

The ANOVA revealed a main effect of Position [F(4,172) = 162.05, *p* < 0.001, *np*^2^ = 0.79], and a significant Position by Cluster interaction [F(8,172) = 13.77, *p* < 0.001, *np*^2^ = 0.39], that reflects different saccade targeting strategy in the 3 three clusters. The effect of Saliency also interacted with Position [F(8,172) = 10.76, *p* < 0.001, *np*^2^ = 0.20]; words with a salient PVL (colored or brighter) attracted more ILPs than a neutral PVL (*p* < 0.002 for P2). Interestingly, the ANOVA also revealed a significant Saliency x Position x Cluster interaction [F(16,344) = 7.13, *p* < 0.001, *np*^2^ = 0.249]. This three-way interaction reflects the differential effects of PVL saliency across clusters, as illustrated in Figure 4. In order to examine the effects of Saliency, Position, and Cluster in more detail, separate ANOVAs were conducted for each cluster.

**Cluster 1**. The ANOVA revealed a large effect of Position [F(4,76) = 122.56, *p* < 0.001, *np*^2^ = 0.87]. Post hoc analyses confirmed that ILP frequency differed significantly across positions (*p*s < 0.01 for all comparisons, except between P4 and P5, *p* = 0.15), reflecting a preference to land on P2, consistent with a PVL effect [26,92] No other effects reached statistical significance.

**Cluster 2**. The ANOVA revealed a main effect of Position [F(4,52) = 85.45, *p* < 0.001, *np*^2^ = 0.85], reflecting a tendency to land on P1 (52%), as well as a significant Saliency x Position interaction [F(8,104) = 13.93, *p* < 0.001, *np*^2^ = 0.52]. As shown in Figure 4, there was a significant effect of brightness on the location of ILPs, resulting in changes in saccade-targeting strategies at P1 and P2 (all *p*s < 0.001), but not at the other three positions (all *p*s > 0.10). Post hoc tests revealed that this interaction stemmed from opposite effects of PVL saliency at P1 and P2: ILP frequency was highest at P1 and decreased at P2 in the neutral condition, whereas ILP frequency increased from P1 to P2 in the brightness condition (all *p*s < 0.001). ILP frequency at P1 and P2 did not differ between the color and neutral conditions.

**Cluster 3**. The ANOVA revealed a main effect of Position [F(4,40) = 18.71, *p* < 0.001, *np*^2^ = 0.65], with no clear evidence of a PVL effect. Here again, a significant Saliency x Position interaction was also observed [F(8,80) = 5.03, *p* < 0.001, *np*^2^ = 0.33]. As shown in Figure 4, there was a saliency benefit on ILP frequency in both the brightness and color conditions, reflected by the emergence of a PVL effect. The saliency effect was significant at P2 (*p* = 0.01), P3 (*p* = 0.02), and P4 (*p* = 0.03) for the brightness condition, and at P1 (*p* = 0.01), P2 (*p* = 0.01), P3 (*p* = 0.02), and P4 (*p* = 0.03) for the color condition.

### 2.7. Number of Fixations

**Figure 5:**
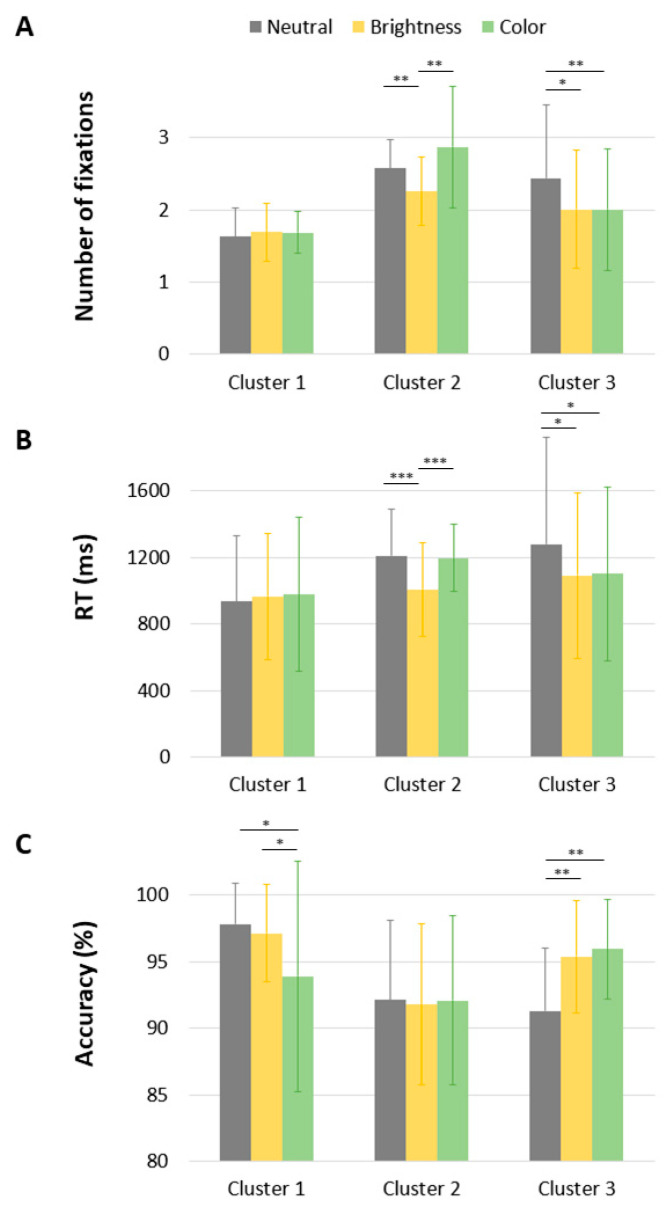
Mean number of Fixations (**A**), RT (in milliseconds) (**B**), and correct answer (in percent) (**C**) as a function of Cluster and PVL saliency. * *p* < 0.05; ** *p* < 0.01; *** *p* < 0.001.

The ANOVA revealed a main effect of Saliency [F(2,46) = 4.92, *p* = 0.009, *np*^2^ = 0.097], indicating that words with a brighter PVL were identified with fewer fixations than words with a neutral or colored PVL (1.98 vs. 2.21 and 2.19, respectively). The analysis also revealed a main effect of Cluster [F(2,46) = 13.56, *p* < 0.001, *np*^2^ = 0.37]. Bonferroni post hoc comparisons showed that the number of fixations in Cluster 1 was lower than in Cluster 2 and Cluster 3 (all *p* < 0.05), while the difference between Cluster 2 and Cluster 3 was not significant. As illustrated in Figure 5, the ANOVA also revealed a significant Saliency × Cluster interaction [F(4,92) = 5.61, *p* < 0.001, *np*^2^ = 0.196]. Whereas PVL saliency had no effect in Cluster 1 (F < 1), it reduced the number of fixations in Cluster 2 and Cluster 3 [F(2,92) = 4.40, *p* = 0.02 and F(2,92) = 4.63, *p* = 0.02, respectively]. Interestingly, post hoc comparisons revealed a significant benefit of brightness only for Cluster 2 (*p* = 0.003 and *p* = 0.01 for the differences between brightness/color and brightness/neutral, respectively; color/neutral *p* = 0.19). In Cluster 3, both brightness and color led to fewer fixations (*p* < 0.001 and *p* = 0.03 for the differences between brightness/color and neutral/color, respectively; brightness/color ns).

### 2.8. Response Time (For Correct Responses Only) (RT)

As detailed in Table 3, a significant main effect of Saliency was observed [F(2,45) = 10.87, *p* < 0.001, *np*^2^ = 0.195]. Post hoc comparisons with Bonferroni correction revealed that reaction times were significantly shorter when the PVL was brighter (M = 1021 ms) than when it was colored (M = 1095 ms, *p* = 0.004) or neutral (M = 1143 ms, *p* = 0.001). The difference between the colored and neutral conditions was not statistically significant (*p* > 0.10). In line with previous findings on the number of refixations, we found a significant Saliency × Cluster interaction on response times [F(4,90) = 6.83, *p* < 0.001, *np*^2^ = 0.23]. For Cluster 1, there was no effect of Saliency [F(2,90) = 1.35, *p* > 0.10]. For Cluster 2, there was a main effect of PVL Saliency [F(2,90) = 32.40, *p* < 0.001], with words presented with a brighter PVL being processed more quickly than those with a neutral or colored PVL (1007 ms vs. 1210 ms and 1195 ms, *p* < 0.001). The difference between neutral and colored PVL was non-significant. We also found a main effect of PVL Saliency in Cluster 3 [F(2,90) = 3.51, *p* < 0.05], with words presented with a salient PVL being processed more quickly than those with a neutral PVL (1101 ms and 1091 ms vs. 1280 ms). The difference between brighter and colored PVLs was non-significant.

### 2.9. Percentage of Errors

The ANOVA revealed a significant main effect of Cluster, F(2, 46) = 4.57, *p* = 0.01, *np*^2^ = 0.167, indicating that participants in Cluster 2 made more errors (8%) than those in Cluster 3 (5.8%) and Cluster 1 (3.7%). Post hoc comparisons showed that the difference between Cluster 2 and Cluster 3 was significant (*p* < 0.05), while the difference between Cluster 2 and Cluster 1 was not (*p* > 0.10). In addition, there was a significant Cluster × Saliency interaction, F(4,92) = 4.10, *p* = 0.004, *np*^2^ = 0.15. While PVL saliency had no effect on response accuracy in Cluster 2 (F < 1), it significantly affected the percentage of errors in Cluster 1 and Cluster 3, F(2,92) = 4.63, *p* < 0.05, and F(2,92) = 6.34, *p* < 0.01, respectively. Notably, in Cluster 3, salient PVLs, either through brightness or color, reduced error rates (4.1% and 4.6%, respectively) compared to the neutral condition (8.7%). In contrast, for Cluster 1, the presence of a colored PVL increased error rates (6.1%) compared to the neutral (2.2%) and the bright PVL (2.9%) conditions (*p*s < 0.05).

## 3. Discussion

We presented the results of an experimental study designed to evaluate an oculomotor reading aid for beginning readers and for those with pronounced oculomotor and VA deficits. Inspired by the promising findings of [94], who showed that saccade-targeting efficiency can be enhanced through real-time feedback on ILPs, we manipulated the relative luminance of the character located at the PVL. The rationale behind this manipulation was that a brighter or colored character within a word could act as a salient visual cue for the saccadic targeting system—effectively serving as the target of the saccadic eye movement. This, in turn, was expected to support young or inefficient readers in optimizing their saccade-targeting strategies, by shifting their ILPs closer to the PVL.

Firstly, our findings confirm that ILP patterns are indicative of a participant’s reading level and can serve as an index of reading efficiency [23,25,26,109]. In our cohort, we identified three distinct subgroups of readers based on their saccade-targeting strategies. Good readers (Cluster 1) typically fixated near the PVL on their first saccade. In contrast, readers with lower proficiency showed a tendency to fixate near the beginning of the word (Cluster 2) consistent with a more serial, letter-by-letter decoding strategy. Participants in Cluster 3—characterized by strong oculomotor and VA deficits—exhibited highly variable ILPs spread around the center of the word, reflecting imprecise and unstable saccade targeting. As readers gain more exposure to print, they gradually shift from sublexical to more parallel lexical processing, with ILPs progressively approaching the PVL [22,27,110]. Additionally, their VA capacity expands toward the constraints of visual acuity, enabling efficient letter encoding [111]. Our findings also highlight the key role of print exposure in shaping eye movement control in children. Cluster 1, characterized by efficient saccade targeting at the PVL, was mainly composed of adults (66%) and fewer children (34%). In contrast, Cluster 2, marked by fixations near the beginning of words, was predominantly composed of children (79%), with only 21% adults. The present finding of a tendency to initially fixate on word beginnings, followed by a corrective fixation at the PVL, suggests that, at least in a considerable number of cases, serial decoding is still present in adult poor readers. Cluster 3 showed a more balanced distribution, with 58% adults and 42% children. Although we controlled word length, between five and six letters, to vary the saccade size between P2 (for 5-letter words) or P3 (for 6-letter words), one potential limitation is the relatively fixed spatial location of stimuli in the right visual field, which might have encouraged automated saccade programming over repeated trials. Although this predictability was required for our ILP measurement, it could limit the generalizability of the findings to natural reading. Nonetheless, we found consistent differences in landing patterns across clusters, suggesting that participants did not adopt a uniform saccade strategy. If spatial regularity alone had driven behavior, we would have expected more homogeneous landing distributions across participants. However, the persistence of distinct cluster profiles indicates that our paradigm still captured meaningful differences in saccade computation strategies, rather than simply reflecting task-specific adaptation.

Secondly, we demonstrated that enhancing PVL saliency was effective for readers with immature saccade-targeting strategies—characterized by ILPs near the beginning of words and low reading-level scores (Cluster 2), and those with inefficient saccade computation—characterized by flattened and diffuse LP curves, along with VA and oculomotor deficits (Cluster 3). Notably, in Cluster 2, only the brightness manipulation yielded an improvement, with a peak shift in the landing site distribution toward the PVL. One possible explanation for the differential impact of brightness and color manipulations lies in the distinct processing characteristics of peripheral vision. At first glance, the brightness condition (i.e., luminance contrast) is known to be more salient and more easily detected in the visual periphery than chromatic (color) information, due to the reduced density of cone photoreceptors and color-sensitive pathways outside the fovea [108,112]. To test this hypothesis, we computed the contrast ratio between the color condition of the highlighted letter [0, 255, 0] and the color of the other letters in the word [216, 216, 216], which equals 1.18. We also calculated the contrast ratio between the brightness condition [255, 255, 255] and the color of the other letters in the word [216, 216, 216], which equals 1.16. The contrast ratio is almost identical between the two conditions, which does not explain their differentiated effects. Interestingly, after the experiment, almost all participants reported being unaware of the presence of the brightness condition, whereas all participants detected the color condition. It is possible that the color requires additional irrelevant visual processing and may have disrupted the perception of the overall structure of the word, which affects the reading process. This hypothesis is supported by the study by [113], who observed that reading time increases when different colors are used within the word, whether at the letter or half-word level. This suggests that the differential effects of brightness and color cues may reflect fundamental neuro-anatomical and functional constraints of the peripheral visual system.

We also found that the saliency manipulation positively impacted response times (RTs) and the number of fixations per word in less proficient readers and in participants with oculomotor and VA deficits. Both total fixation counts and RTs were higher in Clusters 2 and 3 compared to fluent readers in Cluster 1, consistent with prior evidence suggesting that increased RTs following suboptimal landing positions reflect a corrective process occurring before visual word recognition—aimed at providing high-quality visual input for lexical access (e.g., [27]). Guiding the eyes directly to the PVL reduced the number of fixations and, consequently, gaze durations, thereby speeding up the reading process. This finding aligns with two previous studies reporting significantly faster reading speeds and shorter fixation times following either a 10 min VA training using eye-tracking technology [114] or an 8-week intervention, including saccadic control training [83]. This suggests that PVL saliency can improve the saccade-targeting strategy for RD children, and encourage the systematic use of the lexical route, thus providing promising insights for therapists. As with landing position distributions, only the brightness manipulation proved beneficial for less proficient readers.

Regarding reading accuracy, only participants in Cluster 3 showed improvements following both the brightness and color manipulations. Contrary to our expectations, no significant benefit was observed for less proficient readers regarding word recognition. These findings confirm that when reading difficulties stem from linguistic sources or, in the case of very early readers, enhancing PVL saliency to guide eye movements is not sufficient on its own to improve reading performance. While this approach effectively enhances the precision of saccade targeting—resulting in fewer refixations and thus shorter gaze durations—it does not address the underlying cause of the reading difficulty in such cases. Interestingly, expert readers showed the opposite effect in the color condition, with increased error rates. We speculate that a colored letter may interfere with whole-word orthographic processing. Supporting this interpretation [115], an eye-movement study of Finnish first and second graders reported that inserting hyphens between syllables slowed reading compared to normal unhyphenated text—even though the use of hyphens to indicate syllable boundaries is commonly used in Finnish reading instruction. The authors argued that hyphens disrupt whole-word orthographic processing. Although syllable-level decoding plays an important role in early phonological development, beginning readers seem to quickly transition toward a parallel, whole-word processing route for more fluent reading. In line with this, a previous study by the same group [116] showed that only the poorest readers benefited from the introduction of hyphens between morphemic units. This may explain why our participants in Cluster 1 did not show any benefit from the color condition in their reading speed or accuracy measures.

Overall, the findings suggest that a visual cue designed to attract the eye to the PVL was sufficient to improve VA capabilities and saccade-targeting strategies in a substantial proportion of participants, leading to better reading performance and faster word identification. The greatest improvements with the two saliency manipulations were found for participants with significant oculomotor and VA deficits. We do not argue that differences in eye-movement behavior cause reading disorders, but rather that deficiencies in saccade control can, for some participants, impair the reading process, regardless of whether the cause of poor saccade control is difficulty in acquiring reading skills or vice versa. These results suggest that guiding the eye towards the PVL could represent a new way to support reading in participants with VA and oculomotor deficits. Our results align with those of [117], who investigated the effects of an oculomotor rehabilitation treatment on improving reading skills in people with visual impairment. Their findings showed that after oculomotor exercises, dyslexic patients with visual neglect regained normal patterns of eye movements, leading to improved reading performance. Ref. [117] concluded that re-learning eye movement control during reading, and the subsequent improvement in reading performance despite existing visual field defects, highlights the importance of accurate and effective eye-movement control in reading (see also [25,26,82,90,107,118] for similar results in other clinical populations). Several limitations of this study should be acknowledged. The primary limitation concerns the generalizability of our findings. Since only brightness appears to lead to faster and more efficient targeting in Cluster 2—while color has the opposite effect—and since no PVL saliency effect was found on accuracy in this group, it is possible that this effect is purely bottom-up, with limited evidence supporting its persistence without such enhancements, or its generalizability to naturalistic sentence reading. A second limitation of the study concerns the use of a black background. This use of a dark background causes the pupil to open more widely to let in more light, which is ideal for eye-tracking data collection but is neither representative of natural reading conditions on paper nor optimal (e.g., [119]). Given that dark targets mobilize more neural resources in the early visual pathway compared to light targets and are processed more quickly [120], it would be relevant to replicate the observed effects here by reversing the colors.

## 4. Conclusions

In this study, we demonstrated that it is possible to enhance eye movement control during reading by guiding the eyes towards a salient PVL. The most significant improvement was observed in the peak of the landing site distribution, which shifted towards the PVL for all participants in Clusters 2 and 3 under the brightness condition. We also found that this saliency manipulation positively impacted response times and the mean number of fixations per word for the majority of participants. However, only those with strong oculomotor and VA deficits showed improvements in word recognition. In these participants, our manipulation—encouraging the systematic use of the lexical route—may have reactivated residual capacities for whole-word lexical reading, even in individuals unable to access fully automatized processing routines. These findings suggest that developing an oculomotor reading aid for individuals with significant oculomotor and VA deficits is a promising approach. Our results could directly inform therapeutic perspectives, providing potential for early educational adaptations and targeted rehabilitative interventions tailored to specific oculomotor and VA deficits. Such an approach aims to reduce the risk of academic failure and its associated psycho-emotional impacts.

## Figures and Tables

**Table 1:** Characteristics, DEM-test, and Alouette test performances of our sample.

	Cluster 1	Cluster 2	Cluster 3
**Demographics**			
N	23	14	12
Participant ratio (Children/adults)	8/15	11/3	5/7
Sex ratio (F/H)	17/23	8/6	5/12
Participant age			
Children	92 (5.2)	93 (6.2)	94 (4.0)
Adult	246 (18.4)	249 (26.5)	246 (12.7)
**DEM-test**			
VT (*Z-score*) (SD)	0.440 (0.633)	0.029 (0.577)	−1.414 (13.7)
HTaj (*Z-score*) (SD)	0.052 (0.513)	−0.013 (1.077)	−1.945 (30.9)
**Alouette test**			
Reading Fluency (*Z-score*) (SD)	0.630 (1.060)	−1.261 (0.789)	−0.348 (0.558)

Notes. VT: Vertical Time; HTaj: Adjusted Horizontal Time.

**Table 2:** Mean of initial landing position frequency (in percent), as a function of saliency, position, and cluster.

	ILP Frequency Mean (SD)		
	Cluster 1	Cluster 2	Cluster 3
**Neutral**			
P1	22.8 (0.13)	66.4 (0.19)	17.2 (0.22)
P2	63.2 (0.11)	27.2 (0.08)	40.3 (0.07)
P3	13.2 (0.9)	4.5 (0.16)	35.1 (0.20)
P4	1.0 (0.02)	1.6 (0.06)	7.1 (0.08)
P5	0.0 (0)	0.0 (0)	0.0 (0)
**Brightness**			
P1	23.9 (0.21)	42.0 (0.19)	18.3 (0.17)
P2	58.4 (0.17)	53.2 (0.14)	57.0 (0.16)
P3	15.1 (0.16)	4.4 (0.09)	20.1 (0.20)
P4	2.7 (0.06)	0.2 (0.01)	4.6 (0.05)
P5	0.0 (0)	0.0 (0)	0.0 (0)
**Color**			
P1	26.7 (0.14)	58.8 (0.21)	26.3 (0.25)
P2	60.0 (0.13)	36.8 (0.14)	47.1 (0.12)
P3	13.5 (0.13)	4.2 (0.09)	24.4 (0.24)
P4	1.0 (0.02)	0.2 (0.01)	2.2 (0.03)
P5	0.0 (0)	0.0 (0)	0.0 (0)

**Table 3:** Mean number of Fixations, RT (in milliseconds), and correct answer (in percent) as a function of saliency and cluster.

	Number of Fixations Mean (SD)			RT Mean (SD)			Accuracy Mean (SD)		
	Cluster 1	Cluster 2	Cluster 3	Cluster 1	Cluster 2	Cluster 3	Cluster 1	Cluster 2	Cluster 3
Saliency									
Neutral	1.63 (0.39)	2.57 (0.40)	2.44 (1.01)	939.91 (132.44)	1209.95 (153.33)	1280.31 (255.13)	97.81 (3.03)	92.13 (5.93)	91.30 (4.69)
Brightness	1.68 (0.39)	2.26 (0.48)	2.01 (0.81)	964.30 (114.30)	1006.95 (138.58)	1090.52 (198.48)	97.13 (3.63)	91.76 (6.05)	95.35 (4.26)
Color	1.68 (0.29)	2.87 (0.84)	2.00 (0.84)	987.98 (132.44)	1195.43 (121.28)	1100.84 (200.04)	93.89 (8.68)	92.09 (6.31)	95.92 (3.75)

## Data Availability

The data presented in this study are available on request from the corresponding author due to confidentiality and privacy disclosure of the participants’ identities and authorized sharing of the data.
